# Framework for Parallel Preprocessing of Microarray Data Using Hadoop

**DOI:** 10.1155/2018/9391635

**Published:** 2018-03-29

**Authors:** Amirhossein Sahlabadi, Ravie Chandren Muniyandi, Mahdi Sahlabadi, Hossein Golshanbafghy

**Affiliations:** ^1^Faculty of Information Science and Technology, Universiti Kebangsaan Malaysia, 43600 Bangi, Malaysia; ^2^Faculty of Creative Multimedia, Multimedia University, 63100 Cyberjaya, Selangor, Malaysia

## Abstract

Nowadays, microarray technology has become one of the popular ways to study gene expression and diagnosis of disease. National Center for Biology Information (NCBI) hosts public databases containing large volumes of biological data required to be preprocessed, since they carry high levels of noise and bias. Robust Multiarray Average (RMA) is one of the standard and popular methods that is utilized to preprocess the data and remove the noises. Most of the preprocessing algorithms are time-consuming and not able to handle a large number of datasets with thousands of experiments. Parallel processing can be used to address the above-mentioned issues. Hadoop is a well-known and ideal distributed file system framework that provides a parallel environment to run the experiment. In this research, for the first time, the capability of Hadoop and statistical power of R have been leveraged to parallelize the available preprocessing algorithm called RMA to efficiently process microarray data. The experiment has been run on cluster containing 5 nodes, while each node has 16 cores and 16 GB memory. It compares efficiency and the performance of parallelized RMA using Hadoop with parallelized RMA using affyPara package as well as sequential RMA. The result shows the speed-up rate of the proposed approach outperforms the sequential approach and affyPara approach.

## 1. Introduction

Thousands of genes are expressed through microarray. The abundance of produced messenger RNA (mRNA) for the expressed genes can be studied using microarray-based methods where it allows large-scale analyses of gene expression simultaneously [1].

Microarray technology enables physicians to compare the expression and regulation of thousands of genes simultaneously and recognize the disease and the ill gene [2].

Microarray data contains noises. It has been distinguished by a high dimensionality. The first step of microarray experiments is significant as it prepares clean data for downward analysis. The preprocessing procedure for the raw microarray data consists of background correction, normalization, and summarization. Afterward, high level analyses such as gene selection, classification, or clustering are executed to profile gene expression patterns [3].

The main reason of microarray data classification is to create a classifier to classify new data and predict the future trend of data [4].

Microarray data preprocessing identifies noise data and eliminates or reduces the impact of existing noises on the machine learning algorithm.

Preprocessing consists of three steps: background correction, normalization, and summarization [5]. Well-known algorithms of microarray data preprocessing are MAS4.0, MAS5.0, RMA, and GCRMA. However, these algorithms are all implemented in a conventional single thread programming. Parallelizing of these algorithms can help to speed up the performance of the preprocessing stage.

Parallel architectures (e.g., multicore systems, GPU, and CELL processors) associated with current programming models (e.g., Service Oriented Architecture, MapReduce) can transform single thread program to multithread program. Hadoop is a distributed file system framework that uses the MapReduce model in order to distribute the jobs across different nodes and then collect the results from nodes and merge them. However, Hadoop always suffers from lack of powerful statistical tools or techniques [6].

As a result, the powerful statistical tool is needed to integrate with Hadoop. In this research, R is chosen to integrate with Hadoop. R is suite of software facilities for data manipulation, calculation, and graphical display [7]. Integrating R with Hadoop facilitates programing MapReduce jobs in R language by Hadoop's streaming API. In this regard, there is a framework called RHadoop, which consists of three packages such as rmr2, rhdfs, and rhbase. These packages ease managing, distribution, and analysis of data with Hadoop [8].

In this research, new approach has been proposed to use RHadoop framework to preprocess microarray cancerous breast data using RMA algorithm. It will increase the preprocessing speed of microarray data while amount of data increases. As a result, the time required for preprocessing decreases considerably. To the best of our knowledge, it is the first time that RHadoop is applied in bioinformatics.

A DNA microarray is a solid surface (i.e., glass) with many DNA spots attached to it. Each spot contains a short sequence of DNA (gene) of interest named probes. Set of probes, which have the same nucleotide sequences, are called probe set that helps to detect the expression of particular gene. Typically, there are two different kinds of probe: Perfect Match (PM) and Mismatch (MM). Each probe is part of the member of probe set with the same nucleotide sequences [9]. Each PM probe is paired with a MM probe. The PM probe represents a part of gene sequence. MM probe and PM probe have similar sequences except MM probe's central position (13th base of 25 probe bases) that is substituted by another base (A → T or G → C). The main purpose of MM probe in the microarray is to measure the nonspecific hybridization and background noise [10].

### 1.1. Problem

Accurate and early diagnosis of disease is vital. For this reason, using microarray technology to recognize the disease is widespread. Preprocessing of microarray data is the most significant step in analyzing of data as any error in this step would lead to wrong result in the whole system. Thus, it is necessary to have a solid plan and method to refine data and make them applicable for further processes. Most of the preprocessing methods are time-consuming. Moreover, traditional sequential microarray quality assessment and preprocessing tools are not able to handle large amount of dataset. Therefore, there is a need of marvelous technologies and techniques to preprocess huge amount of microarray data. One of these techniques is MapReduce programing model that takes advantage of data locality designed to address data intensive problems [11].

## 2. Background Study

### 2.1. Preprocessing

**Figure 1:**
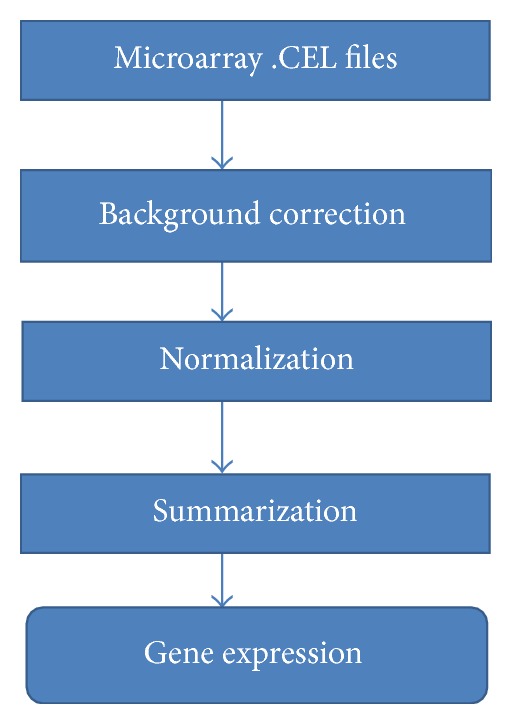
Preprocessing steps [12].

Preprocessing is the most important stage in terms of feeding clean data to the downstream analysis such as gene selection, clustering, and classification. Preprocessing removes systematic errors between arrays. Fundamentally, preprocessing aims to find the differentially expressed genes among arrays and within an array. Naturally, each gene must perform the same in an equal situation. However, there are many environmental factors (amount of sample, room temperature, hands germs, and so on) that cause the same gene expressions differentially. Preprocessing ensures that similar genes among various arrays and within an array are expressed equally even if some environmental factors caused them to be expressed unequally [13]. Those genes that are still expressed differentially after preprocessing are called “genes are gone bad.” Figure 1 is the procedure of preprocessing microarray data.

There are several well-known algorithms for preprocessing of microarray data, MAS.04, MAS.05, RMA, GCRMA, fRMA, and UPC.

MAS.04 and MAS.05 are merely applicable for a single chip. They normalize arrays individually without ability to process multiple arrays at the same time. Besides, both algorithms depend on MM binding which is of low level of intensity and do not clear out all the noises [14]. RMA was introduced to resolve existing problems in the above-mentioned methods. It assumes that the PM intensity is noise background. RMA uses quintile normalization. It results in equal distributions for the probe intensities of every microarray and thus makes their values comparable [10]. Scientists believe that gcRMA's background adjustment introduces more noise than RMA into typical noisy chips produced in the lab [15]. UPC is another famous method proposed after fRMA to remove the dependency on the platforms for conducting experiments. Even though UPC is platform independent, it only determines the probability of the gene expression which is not reliable enough in comparison with other methods' exact value. As a result, RMA still keeps its superiority over other methods. In addition, it is the most common algorithm that has been used during the past decade due to the accuracy and precision of this algorithm.

Below is the description of RMA in detail as it is leveraged in the experiment.

#### 2.1.1. Robust Multiarray Average (RMA)

The RMA algorithm was proposed in 2003 [16] and yet it is the most common and standard algorithm that exists for preprocessing of the data. It no longer depends on mismatch for eliminating the noise and it can process many arrays at the same time with high accuracy and precision. It assumes that the PM intensity is noise background bg plus biological signal *s*.

Perfect  match intensity
  (1) $$\begin{array}{c} {PM}_{ijn}={bg}_{ijn}+s_{ijn}. \end{array}$$

Let *i* ∈ 1,…, *I*, *I* being the microarray wherein probe *j* ∈ 1,…, *J* in probe set *n* ∈ 1,…, *N*. The purpose of background correction is to obtain the value of *s* as noisy PM is the only available value; it can be found by using the formula below:

PM background signal
  (2) BPMijn=Esijn ∣ PMijn>0bg~Nμ,δ2,s~exp⁡ʎ.

Suppose that the distribution of the background noise is normally distributed and also the distribution of the signal is exponentially distributed. Those assumptions make it possible to result in a formula for *E*(*s*_*ijn*_∣PM_*ijn*_) [17].

  PM signal formula
  (3) $$\begin{array}{c} E\left(\;sPM\;\right)=a+b\frac{\emptyset \left(a/b\right)-\emptyset \left(\left(PM-a\right)/b\right)}{ɸ\left(a/b\right)+ɸ\left(\left(PM-a\right)/b\right)-1}, \end{array}$$

where *a* = PM − *μ* − *δ*^2^ʎ, *b* = *δ*, *ɸ*(·) being the standard normal distribution and *∅*(·) the standard normal density function. After this equation, the background corrected value is obtained.

After all data are background corrected; then they should be normalized across microarrays to make them comparable. RMA uses quintile normalization. It results in equal distributions for the probe intensities of every microarray and thus makes their values comparable [10].

Eventually, all the probe intensities' values belonging to the specific probe must be summarized to only one single value. The normalized and background corrected probe intensity *Y* can be illustrated as the true gene expression *θ* plus an effect specific to the probe *∅* and a measurement error [16, 18].

True gene expression
  (4) $$\begin{array}{c} Y_{ijn}=\theta_{in}+\emptyset_{jn}+\in_{ijn}. \end{array}$$

The idea is to estimate the values of *θ*_*in*_, in which the true gene expression values are. Median polish is applied to estimate the gene expression. It helps to find the error (∈_*ijn*_) and then deduct it from *Y* to find the gene expression. Median polish is reliable method against outliers [10].

### 2.2. Parallel Preprocessing

During the last decade, the volume of the microarray data has been increased dramatically as scientists obtain huge amount of data from the daily routine experiment conducted in labs. In parallel processing, a process is divided to several smaller parts and each part is executed at different node separately and simultaneously. Hence, it is much faster and efficient compared to single node processing [19]. Parallel processing can lead to efficiently storing, managing, and manipulating data. As the size of the experimental data is expanding, it becomes cumbersome to manage, store, and analyze the data. High performance computing plays significant role in all the phases of life sciences research pipeline to manage the raw data and process them accordingly.

An approach named master/slave was introduced to handle this large volume of data [20]. Master node invokes worker nodes and sends them copy of dataset attached with list of probe sets. Thus each node performs normalization and summarization using Affymetrix Power Tools (APT) and returns the result to the master node. Eventually, master node compiles all results obtained from different nodes and writes them in a single matrix. However, this architecture requires developers to install APT on each worker node which is time-consuming and tedious. Moreover, the resource and task management is not sufficiently effective due to simple load distribution strategy which assigns each node the same number of jobs.

Micro-CS (Microarray  .CEL file Summarizer) [21] is the tool that automates the preprocessing pipeline. It is a distributed tool that utilizes web services and automatically processes normalization and summarization. Then it collects all related and updated libraries. But this tool is executed sequentially. So it has no considerable effect on the performance of data preprocessing.

Cloud computing can conduct data preprocessing in a parallel way. It provides on-demand access to the shared pool of computer resources (e.g., networking, storage, memory, and processor). Cloud computing is a model that helps companies to share resources and reduce the cost [22]. Bioinformatics applications and tools can be deployed on cloud. There are two available bioinformatics datasets in Amazon EC2 public repository. First one is Annotated Human Genome Data provided by ENSEMBL and the other one is UniGene provided by the National Center Biotechnology Information. Dudley and Butte declare that computational power of cloud computing can be used to analyze the bioinformatics data. Recently, cloud-based platforms for biological applications are being used in research works [23]. Nowadays, Galaxy Cloud is a popular platform which is offered by Amazon Cloud. It is named Platform as a Service (PaaS) and it allows everyone to analyze data in large scales with their computational resources. Cloud4SNP is offered as a Software as a Service (SaaS) by Microsoft Azure as a private cloud. It utilizes data parallelism and applies optimization techniques through filtering of probes with similar Single-Nucleotide Polymorphism (SNP) distributions [24]. However, cloud computing presents several issues regarding the security and privacy of data that are particularly important when analyzing patients' data [25], because patient personal data can be leaked out in cloud computing. These issues induce cloud computing inappropriate for microarray data preprocessing.

There is a new parallel platform based on a multithreaded master/slave architecture proposed in 2016 called ParDMET-Miner. The Master Thread (MT) is responsible for partitioning and distributing the load to each slave thread and collecting results from each node. Slave threads (ST) compute locally the association rules [26]. In spite of the improvement obtained in data preprocessing speed, the problem of high number of candidates for possible polymorphism in 255 genes remains challenging.

Another proposed approach is combining GPU with Hadoop to process the large amount of microarray data in a parallel manner [11]. They have used java language to implement their proposed solution. However, GPU core is much slower than CPU core and they are not supported by many modern operating systems (OS) as they do not contain most of new features of current OS. They are suitable for the video games and physics simulations that require high graphics. Besides, even though java provides great environment to develop different tools, it is not great for in-depth mathematical and statistical analyzing. On the other hand, R is a language that is mainly developed via statistical and mathematical analyses.

This research proposed a new approach exploiting RHadoop framework to preprocess microarray cancerous breast data by RMA algorithm. It increases the preprocessing performance of microarray data especially in big data. It is the first time RHadoop is applied to preprocess microarray cancerous breast data.

### 2.3. Hadoop

Hadoop is a framework built by Google to process huge volume of data exposed to many changes frequently. The key attribute of Hadoop is reliability and redundancy. In case one of the nodes fails, it automatically replicates the data to another machine to avoid missing data. It is easy to write, test, and run the distributed application on one machine where Hadoop scales are of the same code as the other machines. Ultimately, it is economical as it runs on commodity hardware without a need to buy any expensive hardware. Hadoop consists of three main pieces as follows [27]:

- MapReduce: it manages the processing part of Hadoop
- Hadoop Distributed File System (HDFS): it is responsible for managing and distributing files across the nodes
- YARN: it is a framework that assigns available resources to the jobs and tasks.

### 2.4. Parallel Processing in R

R tool was primarily developed to calculate and analyze data by aid of statistic and mathematical equation. It is confined to processing and managing limited size of data. On the other hand, Hadoop is one of the famous and ideal distributed file system frameworks which has high capability of processing large volumes of data with high performance, while it is still immature in terms of statistic and mathematical calculation. By R and Hadoop integration, the shortcomings of both are defeated. Meanwhile, it is cost-effective compared to the other solutions (e.g., supercomputing hardware).

#### 2.4.1. RHadoop

RHadoop is a framework that consists of set of packages: rmr2, rhdfs, and rhbase. These packages facilitate management and distribution of data with Hadoop through R. Below is a brief explanation of RHadoop packages [28]:

- *rmr2*: this package translates R language to the language which complies with MapReduce jobs
- *rhdfs*: this package contains some APIs to manage and control the data in HDFS. This package enables to read from HDFS and store to R data frame. It also writes data from R data frame into HDFS storage
- *rhbase*: the primary purpose of this package is to manage the database for Hbase stores, instead of HDFS files. It provides an R language API as well.

## 3. Methodology

The proposed approach distributes the microarray data over nodes by Hadoop HDFS. Then it runs the preprocessing method in the model of MapReduce programing to propagate the jobs and tasks across all nodes. Consequently, preprocessing performance of microarray data increases.

Affy-library and RHadoop framework make Hadoop HDFS accessible. The  .CEL files along with  .cdf files (chip description file) which are uploaded from local machines are placed into HDFS which is located on Hadoop server. Then, Hadoop propagates the data across DataNodes in a form of blocks automatically. Also the addresses of each block beside other specification of blocks are stored in the Namenode. The  .cdf file contains the necessary information about the  .CEL files such as genotyping, sequencing, and position information of the probes.

Yarn component is the resource manager in Hadoop which automatically handles available resources and jobs. It provides a resource to the application based on the request. Resource manager controls resources according to the reports received from Node Manager (NM) existing in each DataNode.

In preprocessing, the mapper function calculates the background corrected value and then normalizes the data for each DataNode in parallel. Reducer function summarizes all probe intensities' values from particular probe in different arrays to only one single value. Mapper and reducer functions are developed and customized based on requirements stated in Section 2.1.1.

Finally, the result is sent back to the Namenode. This result is read from HDFS into computer file system.

**Figure 2:**
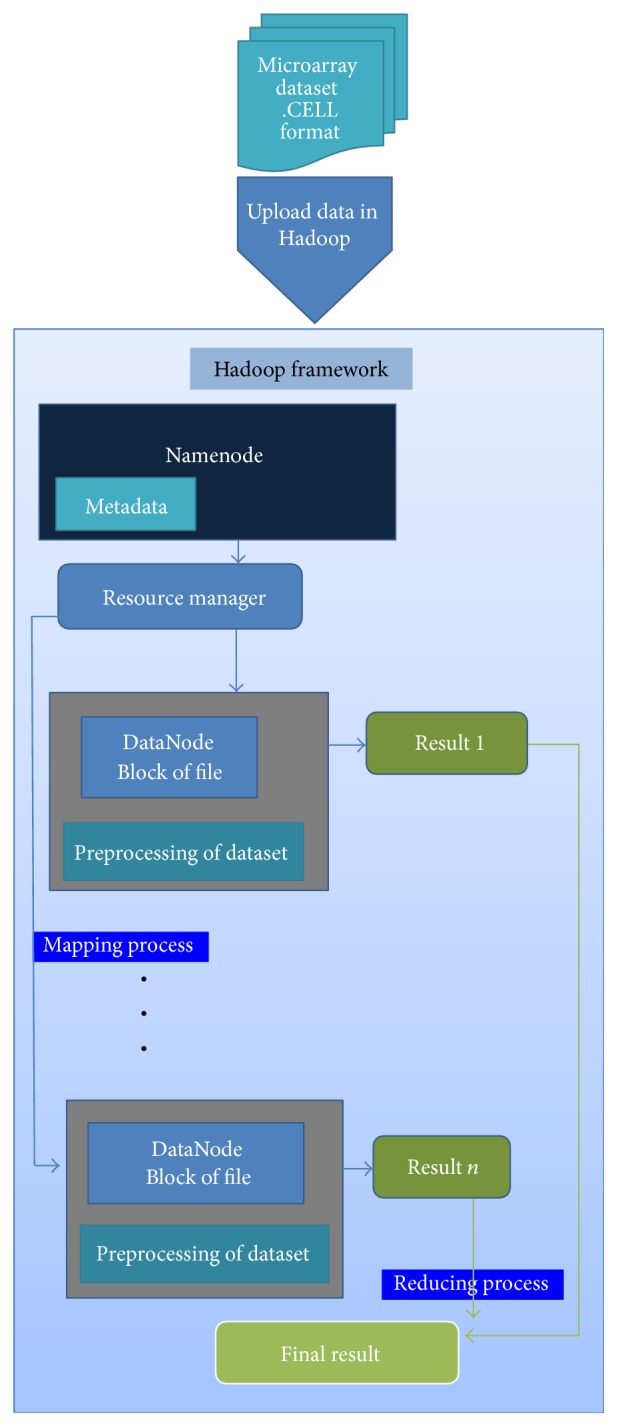
Proposed algorithm.

Figure 2 is the diagram indicating main steps of proposed method. It elaborates how components contribute to data and jobs in each step. It pictures the whole framework.

### 3.1. RHadoop

**Figure 3:**
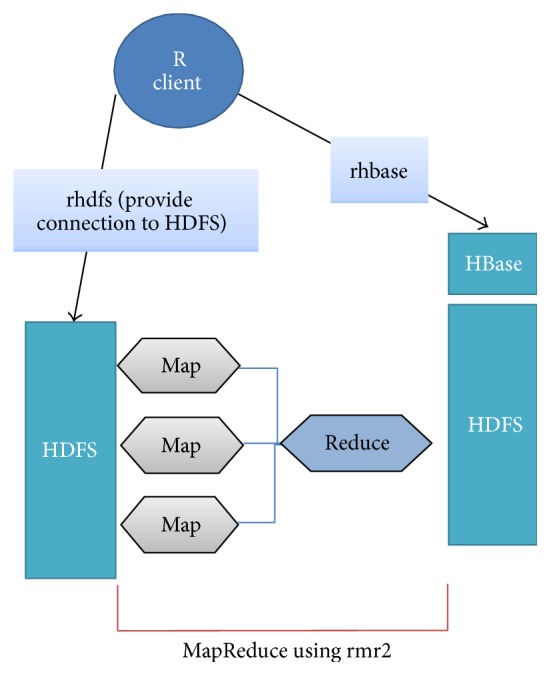
RHadoop architecture.

RHadoop allows developing in R language on Hadoop.  Figure 3 describes how Hadoop components are accessible via R language. RHadoop framework consists of rhdfs, rhbase, and rmr2. The files in the HDFS can be managed and stored by rhdfs [29]. rmr2 package converts existing algorithm in R to MapReduce programing model. So the algorithms run in parallel. Finally, rhbase enables the developer to access the tables to manipulate the records such as read, write, and modifying in Hbase [30].

### 3.2. RMA Implementation in the Proposed Framework

**Figure 4:**
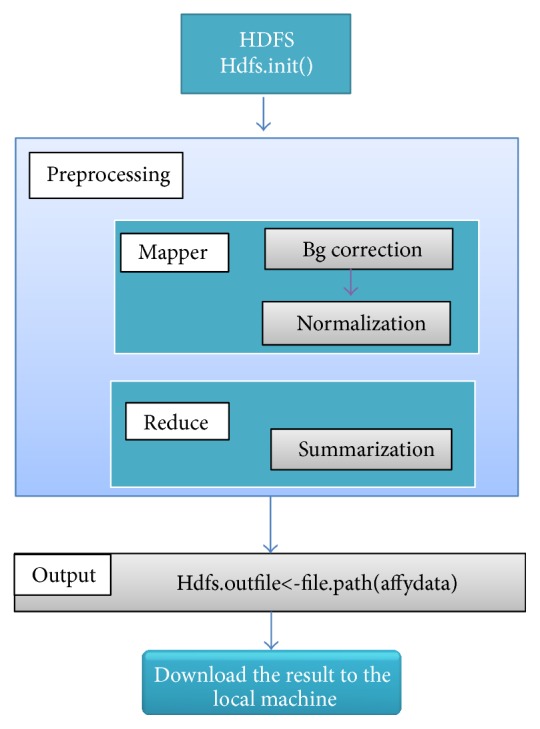
RMA implementation in Hadoop.

Hadoop's components such as YARN and HDFS ON start working by* start-all.sh* command. Afterward, the data must be uploaded from local machine to Hadoop server. Then, Hadoop will propagate the data across different DataNodes in the form of blocks automatically and the addresses of the blocks, along with other specifications of blocks, are stored in the Namenode. To upload the data in Hadoop via R, RHadoop package must be installed in R and then loaded to HDFS library. We need to set the environment variables according to the Hadoop directories and configurations too. Eventually, HDFS must be initialized to work as shown in Figure 4.

After that, rmr2 library is loaded in R to apply MapReduce model of Hadoop to our sequentially implemented RMA. According to Figure 4, the mapper function is written to calculate the background corrected value as well as normalized value of the probs. Reducer function is implemented to summarize the normalized values for the same probe to the single value in different arrays.

Below is the code snippet for mapper and reducer functions which are written in R language.

*Mapper Function*

  map <- function() {
   
-hdfs.get(“hdfs://localhost:9000/affydata/”, “/home/hduser/R/data/hdfstest”)
   
-data<-ReadAffy()
   
-bgdata<-bg.correct.rma(data)
   
-normdata<-normalize.AffyBatch.quantiles(bgdata)
 
 }

*Reducer Function*

  reduce <- function(){
    esetExp <- expresso (normdata, bg.correct = FALSE, bgcorrect.method = NULL, normalize = FALSE, normalize.method = NULL, pmcorrect.method = “pmonly”, summary.method = “medianpolish”)
    write.exprs(esetExp, file = “esetExp.txt”)
 
 }

## 4. Evaluation

The dataset used in this experiment is breast cancer data collected from National Center for Biotechnology Information (NCBI). Microarray cancerous data is found in Gene Expression Omnibus Database (GEO) [31]. This database contains genes and microarray as well as various organism datasets. The GEO accession number for this dataset is GSE4922 which includes list of all GSM files from a single experiment. The dataset used in this experiment starts with GSM110625.CEL and ends with GSM111122.CEL. All tumor samples are evaluated on GPL96 and GPL97. GPL stands for GEO Platform which indicates specific type of platform. GPL96 is a GeneChip of Affymetrix Human Genome U133A Array [HU-133A] and GPL97 is a GeneChip of Affymetrix Human Genome U133B Array [HU-133B]. Both GeneChips are manufactured by Affymetrix [32].

This experiment has been carried out on a cluster containing 5 nodes and each node has 8 cores with 16 gigabyte RAM. Operating system is Ubuntu version 16.04.

### 4.1. Result

**Figure 5:**
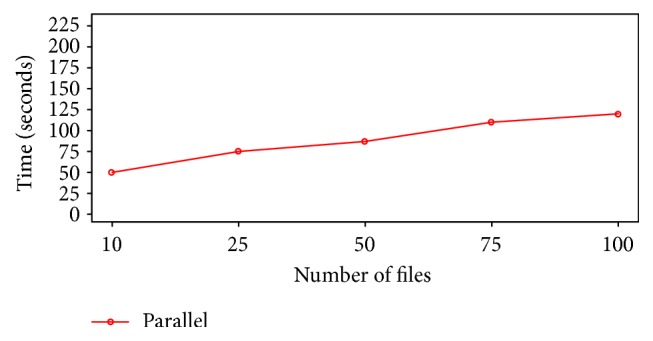
Parallel RMA.

The performance of the proposed approach has been evaluated by measuring the time required to preprocess the data. The specific number of files (10, 50, 100, 150, and 200) is selected and the time for preprocessing of each batch of  .CEL files is calculated. The time to preprocess 10 files is about 50 seconds; when the number of files increases to 50, the time reaches 87 seconds. It means the time's growth is not too much while the number of files increases remarkably. Therefore, the slope is less than 1.  Figure 5 illustrates the speed of the parallel preprocessing of microarray data using Hadoop.

## 5. Discussion

**Figure 6:**
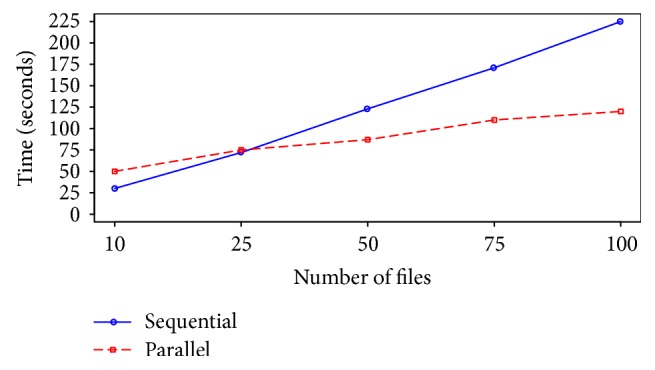
Comparison between parallel RMA and sequential RMA.

Figure 6 compares the parallel RMA approach with the standard sequential RMA preprocessing method. The result shows that as the volume and number of the files increase, parallel preprocessing takes less time in comparison with sequential one.

**Figure 7:**
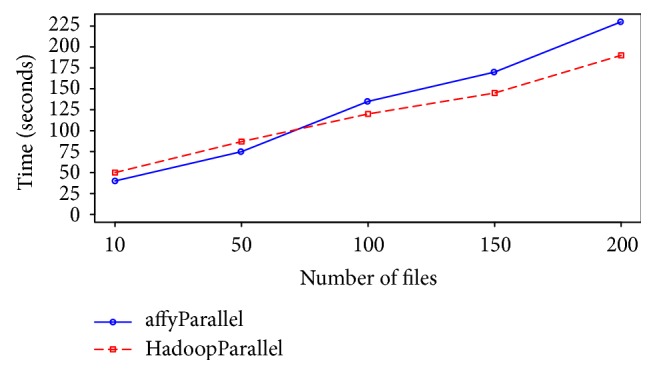
affyPara versus HadoopParallel.

We have also implemented the RMA using preProPara() function in affyPara package and compared the performance with the proposed solution. As it is shown in Figure 7, at first the RMA in affyPara outperformed our solution; however as the number of files increases, the RMA in the proposed framework performs more efficiently. This is because of the Hadoop YARN resource manager that leverages the efficient resource management and job scheduling.

Speed-up rate (ratio of speed in throughput of the proposed approach over the existing approach) has been calculated to show the improvement achieved in speed of the preprocessing of microarray data using our solution. It is observed that as the number of the files increases, the speed increases considerably.

**Figure 8:**
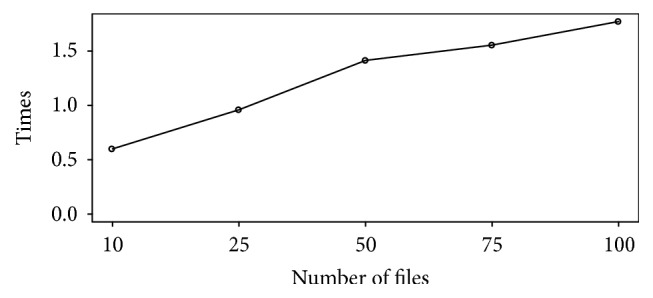
Speed-up rate for parallel preprocessing.

Figure 8 shows that speed-up rate is almost 2 times more than sequential method. Parallel RMA is outperforming sequential RMA, especially when the number of files soars. Although the proposed approach preprocesses data in less time, the biological result is the same as sequential RMA. The speed-up rate has been calculated and plotted on the graph to illustrate the performance of the proposed method over sequential method. Additionally, the proposed approach is also performing better compared to the affyPara package. In addition, the speed-up rate reaches approximately 1.5 for 200 files. The speed-up rate will go up if the number of files increases.

## 6. Conclusion

In this paper, the proposed approach exploits Hadoop and R integration in order to preprocess the microarray data by RMA algorithm in a parallel manner for the first time in bioinformatics. According to the experiment, the result shows performance improvement; as the volume of files increases, it requires less time to preprocess the data compared to the sequential one. Besides, preprocessing of hundreds of microarray datasets using sequential RMA is not possible or, even in some cases, it takes days to accomplish due to its heavy memory usage. The main memory limits are caused by the structure of the* AffyBatch *class. The* AffyBatch *will be created by importing  .CEL files into the R software and is a container for storing probe-level data. The number of arrays which can be imported strongly depends on the architecture of the computer system (e.g., 32-bit Linux system with 4 GB main memory can support 160  .CEL files). The partition of data and distribution to several nodes solves the main memory problems and accelerates the methods [33]. Therefore, the MapReduce implementation of RMA allows processing any size of files conveniently with higher speed. The proposed method has the capability to be implemented in high numbers of clusters with high computational power and memory to handle huge amounts of bioinformatics of data.
